# SNORD1C maintains stemness and 5-FU resistance by activation of Wnt signaling pathway in colorectal cancer

**DOI:** 10.1038/s41420-022-00996-5

**Published:** 2022-04-14

**Authors:** Yonghui Liu, Chengwen Zhao, Guihua Wang, Jing Chen, Shaoqing Ju, Jianfei Huang, Xudong Wang

**Affiliations:** 1 Department of Laboratory Medicine, Affiliated Hospital of Nantong University, Nantong, Jiangsu 226001 China; 2 School of Public Health, Nantong University, Nantong, Jiangsu 226019 China; 3 Department of Clinical Biobank, Affiliated Hospital of Nantong University, Nantong, Jiangsu 226001 China

**Keywords:** Cancer stem cells, Small RNAs

## Abstract

Small nucleolar RNAs (snoRNAs) are a class of non-coding RNAs that play indispensable roles in cancers, including colorectal cancer (CRC). However, the role of SNORD1C in CRC is unclear. In the current study, SNORD1C expression was measured in CRC tissues using quantitative real-time PCR. A series of in vivo and in vitro experiments were performed to examine the functional role of SNORD1C in CRC. Quantitative real-time PCR, western blotting, sphere formation assay, and chemotherapy resistance analysis were conducted to illustrate the SNORD1C molecular mechanism. SNORD1C was upregulated in CRC and that high SNORD1C expression was related to poor prognosis. After knocking down SNORD1C in CRC cell lines, cell proliferation, colony formation, cell migration, and invasion were alleviated, while apoptosis was increased. Transcriptional RNA-sequencing analysis revealed that following SNORD1C knockdown, β-catenin was downregulated, as was the transcription factor TCF7, which inhibited the Wnt/β-catenin pathway. Meanwhile, levels of the stem cell-related factors were reduced, diminishing cell stemness and tumorigenesis. Our findings suggest that SNORD1C functions via the Wnt/β-catenin pathway to enhance cancer cell stemness in CRC and could be a predictive biomarker for the prognosis ad aggressiveness of this malignancy. Additionally, targeting SNORD1C may be a novel therapeutic strategy for CRC.

## Introduction

Colorectal cancer (CRC) is a highly heterogeneous tumor of the digestive system that has ranked second-highest for incidence and third-highest for mortality among all cancers worldwide [1]. Currently, the primary treatments for CRC are surgical resection and chemotherapy. Despite continuous development of anticancer regimens and targeted therapy technologies [2], the high recurrence and low survival rate of patients with advanced-stage CRC remain problems to be solved [3]. Elucidating the underlying molecular mechanisms of CRC progression is extremely critical for the emergence of novel therapeutic strategies.

Small nucleolar RNAs (snoRNAs), are a subset of nuclear non-coding (nc)RNAs with lengths of 60 to 300 nucleotides. According to previous studies, snoRNAs are primarily involved in modifying ribosomal (r)RNAs and small nuclear (sn)RNAs during maturation and processing [4, 5] and are divided into H/ACA box snoRNAs and C/D box snoRNAs [6, 7]. snoRNAs and corresponding proteins form snoRNA-protein (snoRNP) complexes to avoid digestion by restriction enzymes; snoRNA-interacting proteins also play roles in snoRNA biosynthesis and transportation [8]. Finally, snoRNAs often function via inducing 2′-O-methylation or pseudouridylation of rRNAs.

There is growing evidence that abnormal snoRNA activity is involved in cancer development and prognosis. For example, the H/ACA box snoRNA SNORA23 is overexpressed in human pancreatic ductal adenocarcinoma, where it increases SYNE2 expression to promote cell invasion and metastasis [9]. Additionally, SNORA21 is an oncogene in CRC with poor prognostic implications [10]. The C/D box snoRNA SNORD89 is upregulated in ovarian cancer and overexpressed in ovarian cancer stem cells (CSCs). Meanwhile, high SNORD89 expression promotes increased c-Myc and Notch1 expression, which led to cancer formation and poor prognoses [11]. It has also been reported that SNORD52, which is negatively regulated by Upf1, is highly expressed in hepatocellular carcinoma. SNORD52 overexpression stimulates hepatocarcinogenesis by enhancing CDK1 stability [12]. Multiple studies have shown that snoRNAs are of great significance in tumor genesis, development, and recurrence. As is known, ALDH1 is considered to be one of the markers of tumor-infiltrating cells [13, 14]. Mannoor et al. pointed out that in non-small cell lung cancer (NSCLC), the expression of SNORD1C in ALDH + tumor cells was much higher than that in ALDH- tumor cells, and the ALDH + tumor cell group showed a poor prognosis [15], which not only revealed the promoting effect of SNORD1C in malignant tumors, but also suggested that SNORD1C may be closely related to tumor cell self-renewal and cancer recurrence. However, the role of SNORD1C in CRC remains unclear.

In this study, we analyzed combined data from The Cancer Genome Atlas (TCGA) and GSE76713 database, which demonstrated that the C/D box snoRNA SNORD1C was highly expressed in CRC. Functional in vitro experiments revealed that silencing SNORD1C reduced CRC cell proliferation, invasion, and metastasis but increased apoptosis. Furthermore, knocking down SNORD1C inhibited tumor growth in vivo. Meanwhile, SNORD1C knockdown suppressed Wnt/β-catenin signaling and CSC formation. In summary, our data demonstrated that silencing SNORD1C could decrease CRC development and chemotherapy resistance.

## Results

### SNORD1C was highly expressed in CRC and related to poor prognosis

**Fig. 1:**
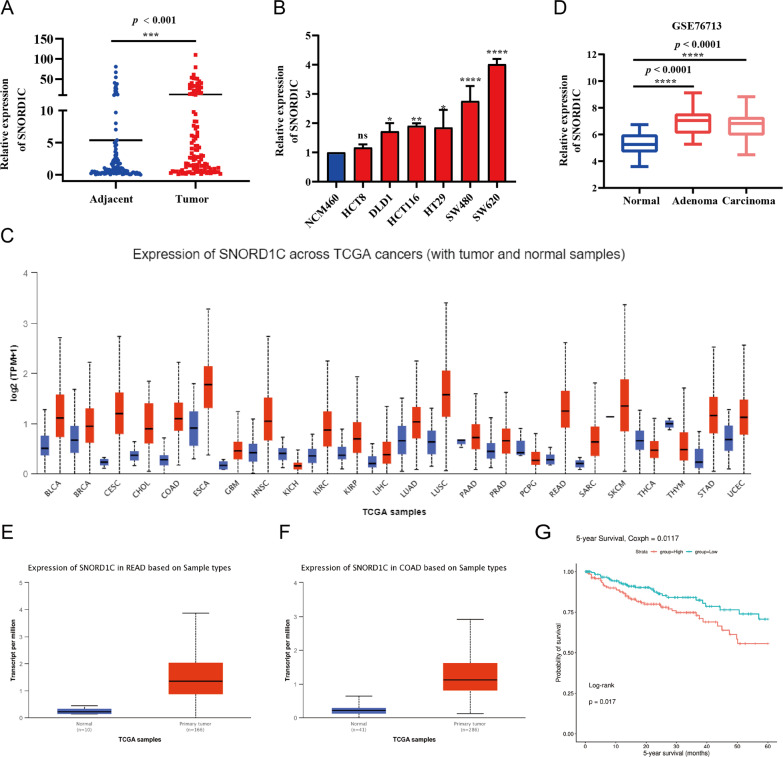
High SNORD1C expression was significantly associated with poor prognosis. **A** SNORD1C expression in 108 paired CRC and paracancerous tissues. **B** SNORD1C expression in NCM460 cells and CRC cell lines. **C** The pan-cancer expression of SNORD1C. **D**–**F** TCGA and GEO datasets were combined to uncover SNORD1C expression in CRC and normal tissues. **G** Kaplan–Meier survival analysis of SNORD1C expression in TCGA. *P < 0.05, **P < 0.01, ***P < 0.001, and ****P < 0.0001.

From our previous study [16], U6 has better linearity and stability than GAPDH, ACTB, TUB, and 18 S for the small nucleolar RNA SNORD1C, and is more suitable as an internal reference for the detection of SNORD1C. Here, to investigate the clinical relevance of SNORD1C in CRC, we measured 108 paired CRC and paracancerous tissues. This revealed that SNORD1C was significantly overexpressed in malignant tissues compared with adjacent tissues, which was consistent with our database analysis (*P* < 0.001, Fig. 1A). Similarly, compared with NCM460 cells, SNORD1C was highly expressed in CRC cell lines (*P* < 0.05, Fig. 1B). Additionally, an analysis of clinicopathological parameters revealed that high SNORD1C expression was related to increased TNM stage (*P* < 0.05) and CEA levels (*P* < 0.05), independent of other factors such as age, sex, and tumor differentiation (*P* > 0.05, Table 1). Then, we examined transcriptome data of CRC cells and tissues from TCGA and GEO (GSE76713) databases. Pan-cancer analysis showed that SNORD1C expression was generally higher in cancers than in paracancerous tissues (Fig. 1C). The findings from TCGA and GEO showed that SNORD1C expression was higher in CRC tissues than in adjacent tissues (Fig. 1D–F). The SNORic database showed that the 5-year survival rate of CRC patients with higher SNORD1C expression was decreased compared with those with lower SNORD1C expression (Fig. 1G). Therefore, SNORD1C could be regarded as an oncogene in CRC that is closely related to poor prognosis.

**Table 1:** Clinicopathological characteristics of CRC patients associated with SNORD1C levels.

Characteristic	Number	Low SNORD1C expression	High SNORD1C expression	*P*-value
Sex				0.100
male	85	39	46	
female	23	15	8	
Age (years)				0.687
<60	38	18	20	
≥60	70	36	34	
Location				0.845
rectum	45	22	23	
colon	63	32	31	
Differentiation				0.260
well	1	1	0	
moderate	97	50	47	
poor	10	3	7	
Size (cm)				0.537
<3	35	19	16	
≥3	73	35	38	
TNM				0.016^*^
I	11	10	1	
II	36	17	19	
III and IV	61	27	34	
Venous invasion				0.197
positive	30	18	12	
negative	78	36	42	
Nerve infiltration				0.156
positive	37	22	15	
negative	71	32	39	
Lymphatic metastasis				0.174
yes	61	27	34	
no	47	27	20	
CEA-blood (ng/mL)				0.033^*^
≤5.0	47	29	18	
>5.0	61	25	36	

Bold entries indicate statistically significant *p*-values.

^*^p < 0.05.

### SNORD1C promoted CRC cell proliferation and cell cycle progression

**Fig. 2:**
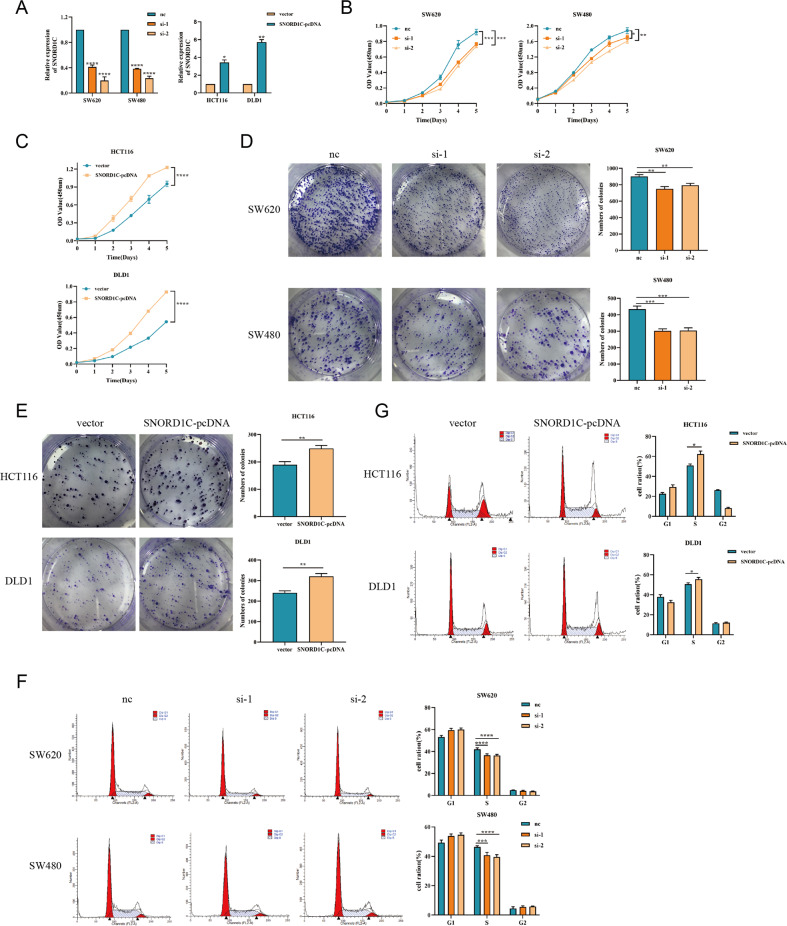
Silencing SNORD1C inhibited the proliferation and self-renewal of CRC cells. **A** qRT-PCR analysis of the efficiency of SNORD1C knockdown (nc, si-1, si-2) in SW620 and SW480 cells and of SNORD1C overexpression in HCT116 and DLD1 cells (vector, SNORD1C-pcDNA). **B**, **C** The CCK-8 assay was used to analyze the proliferation of CRC cells after transfection. **D**, **E** Representative images of colony formation assays are shown to display the proliferation ability of CRC cells after transfection. **F**, **G** Cell cycle analysis was used to assess the self-renewal ability of CRC cells after transfection. All data are shown as mean ± SD; *P < 0.05, **P < 0.01, ***P < 0.001, ****P < 0.0001.

Specific siRNAs (nc, si-1, and si-2) were constructed to knockdown SNORD1C, and then were transfected to SW620 and SW480 cells. Additionally, a SNORD1C plasmid in the pcDNA3.1 vector (vector, SNORD1C-pcDNA) were constructed to overexpress SNORD1C and transfected into HCT116 and DLD1 cells. Transfection efficiency was determined by qRT-PCR (Fig. 2A). CCK-8 assays identified that upregulating SNORD1C significantly promoted cell proliferation, while downregulating SNORD1C had the opposite results (Fig. 2B, C). Moreover, colony formation assays demonstrated that knockdown and overexpression of SNORD1C significantly decreased and increased colony formation ability, respectively (Fig. 2D, E). Cell cycle progression was measured by flow cytometry, and this analysis showed that SNORD1C silencing increased G0/G1 phase and decreased S phase, but had no significant effect on G2/M phase in SW620 and SW480. Ectopic SNORD1C in DLD1 and HCT116 mainly induced significant increases in the proportion of S phase (Fig. 2F, G). Overall, these data suggested that SNORD1C played a key role in accelerating tumorigenesis by promoting cell proliferation and cell cycle progression.

### SNORD1C inhibited apoptosis and promoted migration and invasion in CRC cells

**Fig. 3:**
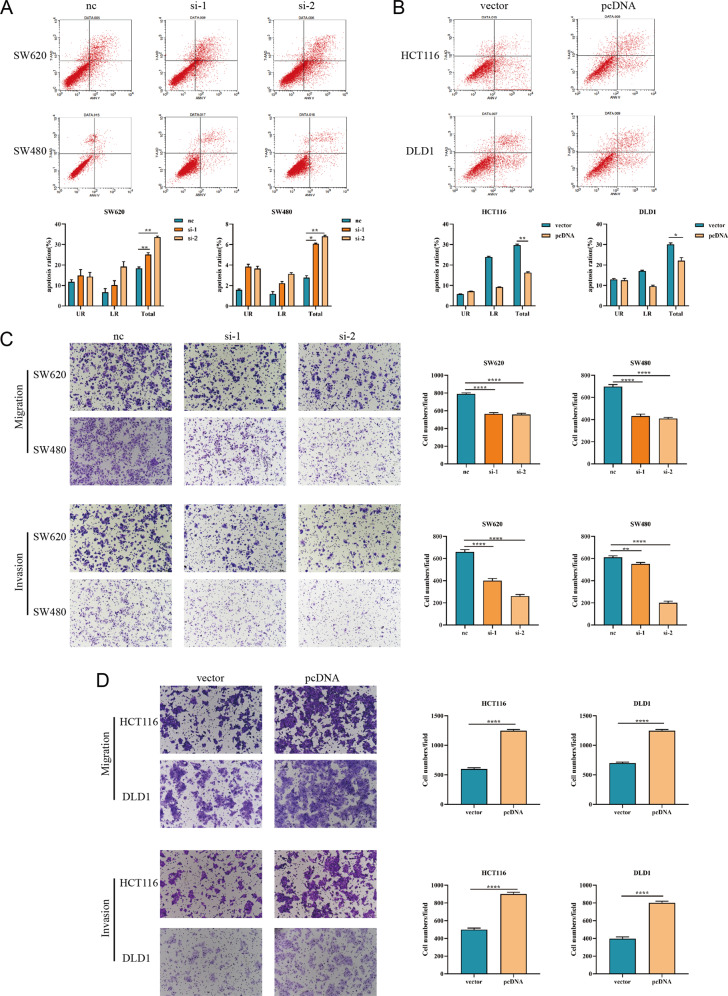
Knocking down SNORD1C promoted apoptosis and inhibited the migration and invasion of CRC cells. **A**, **B** Flow cytometric analysis was conducted to evaluate the effect of knocking down and overexpressing SNORD1C in CRC cells on apoptosis. **C**, **D** Representative images of the cell migration and invasion assays are presented following SNORD1C knockdown or overexpression. All data are shown as mean ± SD; *P < 0.05, **P < 0.01, ***P < 0.001, ****P < 0.0001.

We next examined the influence of SNORD1C on CRC cell apoptosis using the Annexin V PE and 7-AAD assay. Flow cytometry results revealed the changes in early and late apoptotic CRC cells after transfection, and the total apoptotic rate was calculated. In SW620 and SW480 cells with SNORD1C knockdown, the early, late, and total apoptosis rates were all increased (Fig. 3A). In contrast, in HCT116 and DLD1 cells, the apoptosis rates were all decreased following SNORD1C overexpression (Fig. 3B). To detect the effects of SNORD1C on the migration and invasion of CRC cells in vitro, we next performed transwell assays. Cell migration and invasion were significantly decreased in SNORD1C knockdown groups compared with controls. Reciprocally, transwell assays demonstrated that overexpressing SNORD1C increased the migration and invasion abilities of CRC cells (Fig. 3C, D).

### Knocking down SNORD1C suppressed the tumorigenesis of CRC cells in vivo

**Fig. 4:**
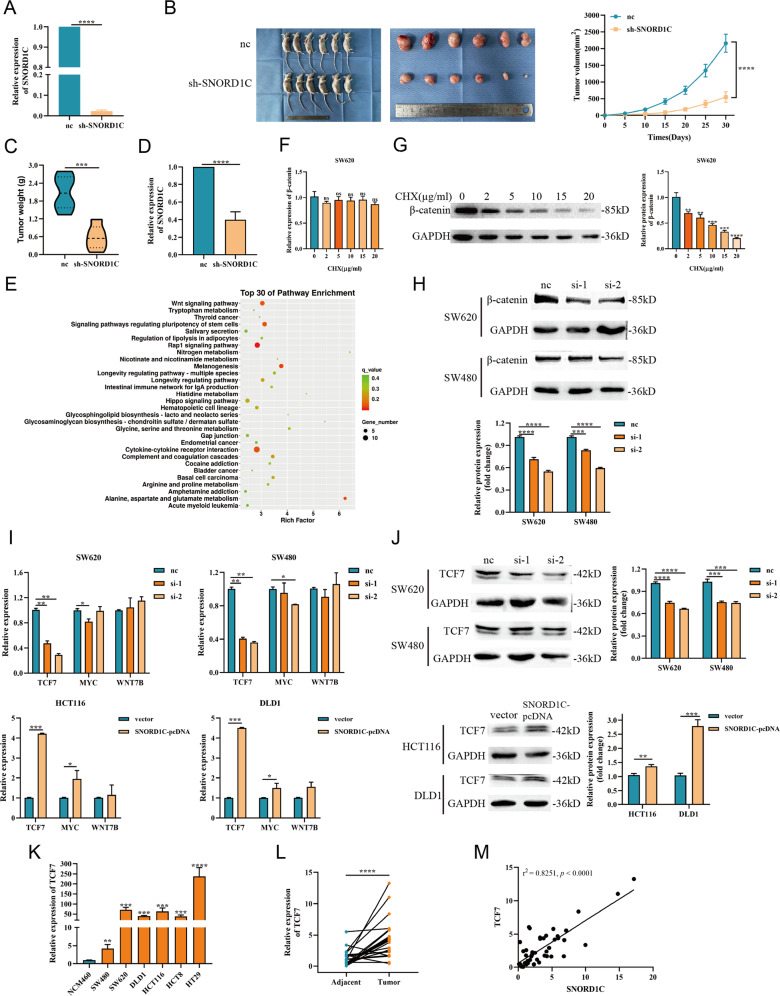
SNORD1C altered the carcinogenesis of CRC cells in vivo, and knocking down SNORD1C inhibited activation of the Wnt/β-catenin pathway. **A** qRT-PCR was used to detect the expression of SNORD1C after lentiviral infection with nc and sh-SNORD1C. **B** Representative images of tumors in nude mice xenograft tumor models and tumor growth curves between nc and sh-SNORD1C groups. **C**, **D** Comparison of tumor weight and qRT-PCR analysis of SNORD1C expression in the model mice. **E** KEGG analysis of differentially expressed genes after SNORD1C knockdown. The top 30 pathways are shown. **F** The mRNA expression of β-catenin showed no difference with the increase of CHX concentration. **G** The protein expression of β-catenin decreased with the increase of CHX concentration. **H** Western blotting experiments were performed to determine β-catenin expression in SNORD1C-knockdown SW620 and SW480 cells. **I** qRT-PCR was used to detect the expression of Wnt pathway-related genes in SNORD1C-knockdown cells. **J** Western blotting was used to evaluate TCF7 levels in SNORD1C-knockdown cells. **K**, **L** qRT-PCR was used to detect TCF7 levels in CRC cell lines and paired tissue samples. **M** Correlation analysis between SNORD1C and TCF7 expression in paired tissue samples. CHX, cycloheximide, the translational inhibitor.

Considering the crucial roles of SNORD1C in vitro, stable nc- and sh-SNORD1C SW620 cells were constructed and injected into mice. The lentiviral transfection efficiency was detected by qRT-PCR (Fig. 4A). Tumors were measured every 5 days until their removal. Tumors of mice were weighed, and their growth curves were plotted. The results suggested that mice in the sh-SNORD1C group had decreased tumor weight and slower tumor growth compared with those in the nc group (Fig. 4B, C). RNA was extracted from the tumor tissues of mice, and the results showed decreased SNORD1C expression in the sh-SNORD1C group compared with the nc group (Fig. 4D).

### SNORD1C cooperated with TCF7 to activate the Wnt/β-catenin pathway

To investigate the mechanism through which SNORD1C achieved these effects in CRC, RNA-seq was performed to compare the SNORD1C-knockdown group with control SW620 cells. The differentially expressed genes (DEGs) are shown in a scatter plot, volcano plot, and heat map (Fig. S1A–C). KEGG analysis indicated that the Wnt signaling pathway and signaling pathways regulating stem cell pluripotency were key altered pathways in SNORD1C-deficient cells (Fig. 4E). The Wnt/β-catenin pathway has been extensively studied in cancers, especially CRC. Abnormalities in Wnt/β-catenin signaling appear in almost all sporadic CRC cases [17]. Several excellent reviews describe how β-catenin is a key regulator of canonical Wnt signaling [18–20]. In order to explore the underlying mechanism of CRC progression, we studied its transcriptional regulation. β-catenin mRNA and protein levels were altered after treatment with the translation inhibitor cycloheximide (CHX). As can be seen from Fig. 4F, G, mRNA level of β-catenin showed no significant difference, while protein level decreased depending on the increase of CHX in SW620 (please see Supplementary Materials for original western blotting data). Then, we observed decreased β-catenin expression after SNORD1C knockdown in SW620 and SW480 cells, which indicates that downregulating SNORD1C inhibited Wnt pathway activation (Fig. 4H, please see Supplementary Materials for original western blotting data). Along with the sequencing results that produced the DEGs, we simultaneously detected and screened target genes of the Wnt signaling pathway, among which the expression of TCF7, MYC, and WNT7B were detected by qRT-PCR in SNORD1C-knockdown and -overexpressing cell lines. These experiments revealed that after SNORD1C was knocked down, TCF7 was significantly downregulated, and MYC was significantly downregulated in SW620 si-1 and SW480 si-2. The expression of TCF7 and MYC increased significantly after SNORD1C overexpression (Fig. 4I). We then examined the alterations in TCF7 protein levels, which revealed significant changes in TCF7 protein levels that coincided with the previous transcriptional results (Fig. 4J, please see Supplementary Materials for original western blotting data).

It is well established that TCF7 is a β-catenin target gene that regulates Wnt signaling [21]. We detected TCF7 expression in all cell lines, which suggested that TCF7 expression was significantly increased in all CRC cells compared with NCM460 cells (Fig. 4K). In 25 paired CRC and paracancerous tissue samples, TCF7 expression was also higher in malignant tissues than in adjacent tissues (Fig. 4L). Correlation analysis showed that SNORD1C expression was positively correlated with TCF7 expression in CRC (*r*^2^ = 0.8251, *P* < 0.0001, Fig. 4M). Together, these findings demonstrate that high SNORD1C expression in CRC can activate Wnt signaling and promote β-catenin expression, which increases TCF7 expression. In the context of SNORD1C overexpression, increased TCF7 leads to the upregulation of its target gene MYC and accelerated tumorigenesis.

### SNORD1C modulated stemness properties and 5-fluorouracil resistance in CRC cells

**Fig. 5:**
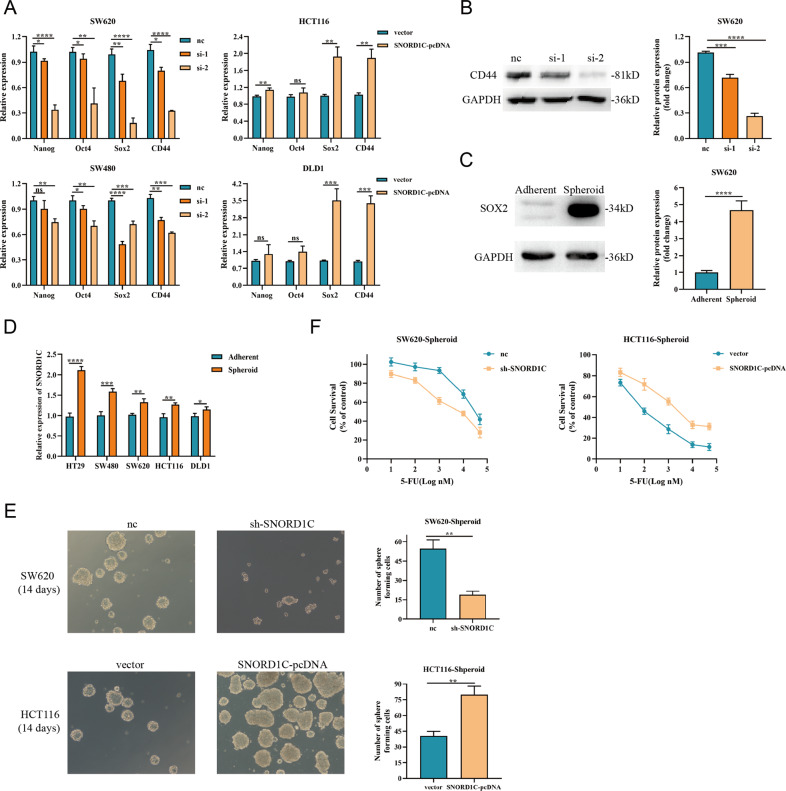
SNORD1C promoted cancer stem cell development and enhanced drug resistance in CRC cells. **A** qRT-PCR was used to determine changes in stem cell regulatory factors after SNORD1C knockdown or overexpression. **B** Western blotting was used to detect CD44 expression after SNORD1C knockdown. **C** Western blotting was conducted to compare SOX2 expression between adherent and spheroid SW620 cells. **D** qRT-PCR was used to compare SNORD1C expression between adherent and spheroid CRC cells. **E** Images of sphere formation in SNORD1C-knockdown and -overexpressing CRC cells. **F** Resistance to 5-FU in SNORD1C-knockdown and -overexpressing CRC cells.

As mentioned above, RNA-seq results revealed that SNORD1C played a vital role in the regulation of pluripotent stemness markers. Several studies have shown that the canonical Wnt/β-catenin pathway is closely related to CSC regulation [22]. TCGA database analysis revealed that SNORD1C was positively correlated with the stem cell regulators OCT4 (POU5F1), NANOG, and LGR5 [23] (Fig. S2A). Thus, we examined multiple stem cell regulatory factors (Nanog, OCT4, SOX2, and CD44), all of which were downregulated in SNORD1C-knockdown cells and upregulated in SNORD1C-overexpressing cells (Fig. 5A). Decreased CD44 protein levels were also observed in SW620 cells following SNORD1C knockdown (Fig. 5B, please see Supplementary Materials for original western blotting data). Thus, we conclude that downregulating SNORD1C regulated CSCs to a certain extent, which led to a decline in their stem cell characteristics.

Therefore, we next performed sphere formation assays in CRC cell lines, which would ascertain the ability of CRC cell lines to acquire stem cell properties. Signature stem cell markers and spheroid enrichment images were used to confirm whether the cells had acquired CSC properties. We observed that SOX2 expression was significantly increased in spheroid compared with adherent SW620 cells (Fig. 5C, please see Supplementary Materials for original western blotting data). Furthermore, SW620 spheres were successfully enriched (Fig. S2B). Next, we constructed spheroid stem cells in several CRC cell lines (HT29, SW480, SW620, HCT116, and DLD1). Notably, SNORD1C expression was higher in spheroid CRC cells than in adherent cells (Fig. 5D). Then, sphere-forming experiments were performed using SW620 cells following stable transfer of sh-SNORD1C and HCT116 cells overexpression SNORD1C. Interestingly, after 14-days serum-free suspension culture, spheroid formation was reduced and irregular when SNORD1C was silenced in SW620 cell; the opposite results were observed in SNORD1C-overexpressing HCT116 cells (Fig. 5E). Moreover, when serum-containing medium was added after sphere formation, CRC cells underwent serum-induced differentiation, which was characterized by sphere dispersal, and the cells beginning to differentiate (Fig. S2C).

It has been demonstrated in a number of studies that SOX2 is highly expressed in many CSCs and associated with chemotherapy resistance [24]. To further investigate the role of SNORD1C in chemotherapy resistance, we treated infected CRC cells with 5-fluorouracil (5-FU). CCK-8 assays showed that the viability of SW620 spheroid cells infected with sh-SNORD1C (IC_50_ = 5.172 µM) was significantly lower than that of control cells (IC_50_ = 30.989 µM). The viability of HCT116 spheroid cells transfected with SNORD1C-pcDNA (IC_50_ = 1874 nM) was significantly higher than that of control cells (IC_50_ = 93.26 nM) (Fig. 5F). Together, these data show that SNORD1C regulates CSC formation and promotes chemotherapy resistance in CRC.

## Discussion

Despite advances in treatment, CRC remains a disease with high morbidity and mortality and a poor prognosis. The Wnt signaling pathway is currently considered one of the key pathways involved in the occurrence of CRC [25, 26]. The classical Wnt/β-catenin pathway promotes cancer progression through altering the transcription of downstream target genes. Meanwhile, CSCs are generally believed to be related to cancer metastasis, recurrence, and drug resistance and are a major difficulty in cancer treatments [27]. Analyses of non-coding small RNAs have been abundant in CRC research, becoming a vital part of cancer research. Therefore, we investigated the potential roles of SNORD1C and found that increased expression of the snoRNA SNORD1C was related to lymph node metastasis, increased CEA levels, and poor prognosis. Mechanistically, SNORD1C activated the canonical Wnt/β-catenin pathway and the expression of pluripotent stem cell markers. Therefore, SNORD1C promotes CRC occurrence and drug resistance.

SNORD1C belongs to a class of snoRNAs, which fall into the greater category of non-coding RNAs [4]. In recent years, myriad reports have found indispensable roles of snoRNAs in diseases [28], such as snoRNAs that control ribosomal RNA modification [29], those that serve as miRNA precursors [30, 31], and those that regulate alternative RNA splicing. snoRNAs influence oncogenesis through the formation of snoRNP complexes [32]. Furthermore, snoRNAs can be used as biomarkers for cancer diagnosis and prognosis and are expected to become potential targets for future cancer therapies [33]. In NSCLC, SNORD1C acts as an oncogenic factor to induce tumor recurrence, leading to low survival rate [15]. However, the role of SNORD1C in CRC remains to be explored. Herein, we found that SNORD1C was upregulated in both CRC tissues and cells and was associated with poor prognosis. Overexpression of SNORD1C in CRC cells promoted cell proliferation, colony formation, invasion, and migration, while inhibiting apoptosis. Allogeneic tumor formation experiments in nude mice showed that SNORD1C-silenced cells grew slowly and had poor tumor formation ability in vivo.

The Wnt signaling pathway is a classic pathway involved in CRC development, and CSCs are the key to accelerating tumor progression [20]. When the Wnt pathway is activated, Wnt ligand interacts with specific receptors on the cell surface, causing the accumulation of β-catenin. Subsequently, free β-catenin can enter the nucleus and regulate transcription in combination with TCF/LEF, thereby altering downstream gene expression, such as c-Myc and cyclinD1 [34]. Several studies have found that stem cell-related regulatory genes such as CD133, CD44, and SOX2 show corresponding changes during tumorigenesis [35, 36]. Wang et al. [37] found that snoU2_19 promoted the Wnt/β-catenin signaling pathway by inducing the nuclear translocation of β-catenin, thereby promoting the progression of hepatocellular carcinoma. Wu et al. [38] also found in hepatocellular carcinoma that SNORD76 could increase the invasiveness of liver cancer cells by inducing epithelial-mesenchymal transition and promote the tumorigenicity of liver cancer by activating the Wnt/β-catenin pathway. CSCs are a major and difficult obstacle to slowing tumor progression, and snoRNAs have been found to be closely related to CSCs. In NSCLC, SNORD78 was significantly overexpressed in cancer stem cell-like cells, which had self-renewal capacity and promoted tumorigenesis [39], and SNORD1C also showed tumor stem cell aggregation in NSCLC [15]. Additionally, SNORA72 was shown to be able to activate the stem cell-like transformation of ovarian cancer cells through the Notch1/c-Myc pathway, which accelerated the occurrence of ovarian cancer [40].

**Fig. 6:**
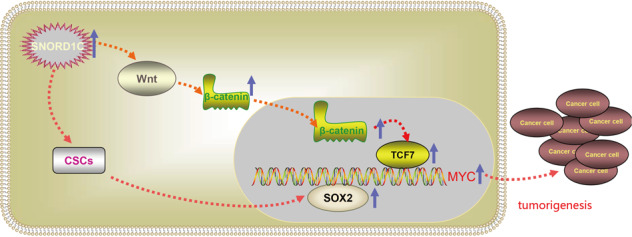
SNORD1C was upregulated in CRC, which activated the canonical Wnt/β-catenin pathway and promoted the development of cancer stem cells. Together, these increased β-catenin expression and that of the transcription factor TCF7, which led to increased expression of the target gene MYC. Furthermore, binding to the stem cell regulator SOX2 promoted cancer development and enhanced chemotherapy resistance.

In this study, we found that SNORD1C acts as an oncogene that regulates the occurrence and development of CRC by promoting cell proliferation, migration, and invasion while inhibiting apoptosis. High SNORD1C expression was related to poor prognosis in CRC patients. We also found that the Wnt signaling pathway and the pathway that regulates pluripotent stem cells were enriched in cells with SNORD1C knockdown using RNA-seq analysis. Silencing SNORD1C reduced β-catenin expression and inhibited activation of canonical Wnt/β-catenin signaling, thus inhibiting expression of transcription factor TCF7. Expression of the target gene c-Myc was downregulated, as were CD44, SOX2, and other stem cell regulatory genes. Together, these alterations reduced the cell’s ability to resist chemotherapy, which led to reduced CRC development, suggesting the clinical utility of targeting this snoRNA during CRC treatment (Fig. 6). However, this study had some limitations. For example, further samples should be collected to analyze risk stratification in different molecular subtypes of CRC.

## Materials and methods

### Cell culture

The human CRC cell lines SW620, SW480, HCT116, DLD1, HT29, and HCT8 and normal intestinal epithelial cells NCM460 were purchased from the Chinese Academy of Sciences Cell Bank (Shanghai, China). NCM460, SW620, SW480, and DLD1 cells were cultured in complete DMEM medium with 10% fetal bovine serum (FBS; Biological Industries) at 37 °C and 5% CO2. HCT116, HT29, and HCT8 were cultured in complete McCoy’s 5 A medium. The culture media was refreshed every 1–2 days.

### Patient specimens

CRC and paracancerous tissue specimens were obtained from CRC patients (*n* = 108) from the Affiliated Hospital of Nantong University; their clinical information was also collected. The affiliated Hospital of Nantong University approved the study. All patients participating in the study obtained informed consent.

### SNORD1C interference, overexpression, and lentiviral transfection

Small interfering (si)RNA against SNORD1C, negative control (si-nc) siRNA, and SNORD1C plasmid in the pcDNA3.1 vector were obtained from GenePharma Ltd. (Shanghai, China). Transient transfection was performed using Lipofectamine 3000 (Invitrogen, Carlsbad, CA, USA) following the manufacturer’s recommendations. The siRNA sequences are listed in Table S1. Briefly, after cells grew to 40–60% confluence in 6-well plates, they were washed with phosphate-buffered saline (PBS) twice, and then SW480 and SW620 cells were transfected with si-nc or si- SNORD1C, DLD1, and HCT116 cells were transfected with empty vector (vector) or SNORD1C-pcDNA. SW620 cells were infected with lentiviral-nc (nc) or lentiviral-RNAi (sh-SNORD1C) (Shanghai Genechem Co., Ltd., Shanghai, China) according to the supplier’s instructions.

### RNA extraction and quantitative real-time (qRT)-PCR

TRIzol (Invitrogen) was applied to extract total RNA from cells or tissues. RNA concentrations and A260/A280 nm ratios were detected using a Nanodrop spectrophotometer (Thermo Fisher Scientific, Waltham, MA, USA). PrimeScript™ RT Reagent (Takara, Shiga, Japan) was used to reverse transcribe cDNA; qRT-PCR was performed by SYBR Green mix (Toyobo, Osaka, Japan). Primer sequences specific for SNORD1C were designed by RiboBio (Guangzhou, China). GAPDH and U6 were used as references for normalization. The relative expression of target genes was calculated by the 2^−ΔΔCt^ method. The primers used for qRT-PCR are listed in Table S2.

### Cell cycle analysis

Cells (1 × 10^6^) were collected by trypsinization 48 h after transfection, washed in cold PBS, resuspended in 70% ethanol, and stored at 4 °C overnight. The second day, the cells were incubated in 100 µL RNase A solution at 37 °C for 30 min. After staining with propidium iodide (PI) at 4 °C for 30 min, flow cytometry was performed on a FACS machine.

### Cell counting kit-8 (CCK-8) assay

Cell proliferation was assessed by the CCK-8 assay (Dojindo, Kumamoto, Japan). At 48 h post-transfection, cells were seeded in triplicate in 96-well plates at a density of 3 × 10^3^ cells per well in 100 µL of medium. After 24, 48, 72, and 96 h, 10 µL CCK-8 was added to each well and incubated for 2 h at 37 °C. Optical density values were measured at 450 nm.

### Colony formation assay

Cells (500 cells/well) were seeded in 6-well plates after transfection. After 10–15 days of culture, colonies were formed. Cell colonies were fixed with 4% paraformaldehyde for 1 h and stained with 0.5% crystal violet for 10 min. Excess staining solution was washed away with PBS.

### Transwell assays

Cell migration and invasion were examined using transwell plates (8.0-μm pore size, Corning Inc., Corning, NY, USA). For invasion assays, 100 µL of Matrigel (BD Biosciences, Franklin Lakes, NJ, USA) was smeared evenly in the upper chamber. At 48 h post-transfection, 3 × 10^4^ CRC cells were resuspended in 200 µL serum-free medium in the upper chamber and 500 μL complete medium containing 20% FBS in the lower chamber. Cells were incubated for 48 h. The number of cells that crossed the membrane was counted using Image J software in three random fields.

### Apoptosis analysis

Apoptotic cells were measured by the Annexin V PE/7-AAD apoptosis detection kit. Cells were washed with PBS and collected into tubes using EDTA-free trypsin. Briefly, 1 × 10^5^ cells were collected and resuspended in 1× binding buffer with 5 μL PE Annexin V and 7-AAD. Then, the cells were incubated at room temperature for 15 min in the dark. Apoptotic cells were identified by flow cytometry.

### Western blotting analysis

Cells were lysed in RIPA buffer containing PMSF, phosphatase inhibitor, and protease inhibitor cocktail. Equal protein amounts were subjected to SDS-PAGE, and then transferred onto PVDF membranes. To prevent non-specific binding, 5% skim milk was applied to block the membranes for 2 h at room temperature. These membranes were then incubated at 4 °C overnight with the following primary antibodies: c-Myc (1:1000, Abcam, Cambridge, UK), β-catenin (1:10000, Abcam), TCF7 (1:1000, Cell Signaling Technology, Danvers, MA, USA), SOX2 (1:2500, Proteintech, Wuhan, China), CD44 (1:1000, Cell Signaling Technology), and GAPDH (1:10000, Proteintech). Secondary antibodies (HRP-conjugated goat anti-mouse or goat anti-rabbit) were obtained from Proteintech. GAPDH levels were used for data normalization.

### Sphere formation assay

HCT116 spheroid cells transfected with empty vector or SNORD1C-pcDNA and SW480 spheroid cells transfected with sh-nc or sh-SNORD1C were seeded onto Ultra-Low Attachment Surface 6-well plates (Corning Inc.) with a density of 2000 cells/well in serum-free DMEM-F12 supplemented with 10 ng/mL bFGF, 20 ng/mL EGF (Sigma-Aldrich, St. Louis, MO, USA), and 2% B27 (Invitrogen). The medium was changed every 3–4 days during sphere formation. After culture for 10–14 days, under an inverted microscope, the number of tumorspheres was counted, and spheres >50 μm in diameter were calculated and plotted.

### Xenograft tumor formation assay

Male 4-week-old nude mice were obtained from the platform of experimental animal center of Nantong University. We used transduced SW620 cells to construct stable transgenic cell lines. Mice were randomly divided into the negative control (nc) group and the sh-SNORD1C group with five mice in each group. Next, 5 × 10^6^ stably integrated SW620 cells were injected into the armpits of mice. Xenograft tumor sizes were measured and recorded on a weekly basis with a vernier caliper. The formula for calculating tumor volume (*V*) was *V* = length (*a*) × width (*b*)^2^ ÷ 2. At 4–5 weeks post-injection, the mice were sacrificed, and subcutaneous tumors were removed and weighed.

### Database analysis

The pan-cancer SNORD1C transcription level was determined using TCGA data (http://cancergenome.nih.gov). Comparisons of SNORD1C in different transcriptome levels in normal, adenoma, and carcinoma tissues were obtained from the Gene Expression Omnibus (GEO) dataset GSE76713. Survival curves came from SNORic (http://bioinfo.life.hust.edu.cn/SNORic/) data [41], and the cohort for survival analysis (*n* = 434) was obtained from TCGA.

### Statistical analysis

Each experiment process was repeated three times. Statistical analyses were conducted using GraphPad Prism software (GraphPad Software, Inc., San Diego, CA, USA). The Student’s *t*-test was applied to assess the difference between two groups. One-way ANOVA was applied to assess differences among more than two groups. Significant differences were considered when *P* < 0.05.

## Supplementary information

Supplementary Material

Original western blots

Cell Line Authentication

## Data Availability

The data that support the findings of this study are available from the corresponding author upon reasonable request.
