# Supplementary material for: SNORD1C maintains stemness and 5-FU resistance by activation of Wnt signaling pathway in colorectal cancer
Source: Cell Death Discov. 2022 Apr 14;8:200. doi: 10.1038/s41420-022-00996-5 (PMC9010412; doi:10.1038/s41420-022-00996-5)
Supplement: Supplementary file 1 — Supplementary Material [file 41420_2022_996_MOESM1_ESM.docx]

**SNORD1C maintains stemness and 5-FU resistance by activation of Wnt signaling pathway in colorectal cancer**

Yonghui Liu, Chengwen Zhao, Guihua Wang, Jing Chen, Shaoqing Ju, Jianfei Huang, Xudong Wang

**Table S1.** SiRNA and LV-RNAi sequences used in this study.

| Gene | Sequences or target sequence (5′-3′) |
| --- | --- |
| SNORD1C-nc | UUCUCCGAACGUGUCACGUTT (sense) |
| SNORD1C-nc | ACGUGACACGUUCGGAGAATT (antisense) |
| si-SNORD1C-1 | GAGCUGAGGAUGAUUUAAATT (sense) |
| si-SNORD1C-1 | UUUAAAUCAUCCUCAGCUCTT (antisense) |
| si-SNORD1C-2 | GGAGGUCUGACUUGCUGAGTT (sense) |
| si-SNORD1C-2 | CUCAGCAAGUCAGACCUCCTT (antisense) |

**Table S2.** Primers for qRT-PCR in this study.

| Gene | Primers (5′-3′) |
| --- | --- |
| TCF7-F | TTGATGCTAGGTTCTGGTGTACC |
| TCF7-R | CCTTGGACTCTGCTTGTGTC |
| MYC-F | GGCTCCTGGCAAAAGGTCA |
| MYC-R | CTGCGTAGTTGTGCTGATGT |
| WNT7B-F | GAAGCAGGGCTACTACAACCA |
| WNT7B-R | CGGCCTCATTGTTATGCAGGT |
| NANOG-F | TTTGTGGGCCTGAAGAAAACT |
| NANOG-R | AGGGCTGTCCTGAATAAGCAG |
| OCT4-F | CTTGAATCCCGAATGGAAAGGG |
| OCT4-R | GTGTATATCCCAGGGTGATCCTC |
| SOX2-F | GCCGAGTGGAAACTTTTGTCG |
| SOX2-R | GGCAGCGTGTACTTATCCTTCT |
| CD44-F | CTGCCGCTTTGCAGGTGTA |
| CD44-R | CATTGTGGGCAAGGTGCTATT |
| GAPDH-F | ACAACTTTGGTATCGTGGAAGG |
| GAPDH-R | GCCATCACGCCACAGTTTC |


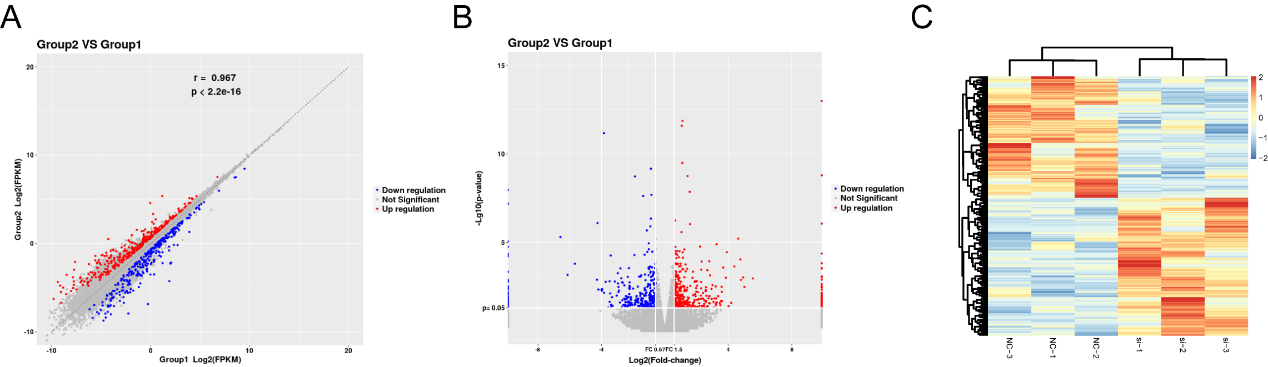


**Fig. S1** **Differentially expressed genes (DEGs) were analyzed by RNA-seq in SW620. A** The scatter plot of DEGs. **B** The volcano plot of DEGs. **C** The heatmap of DEGs.


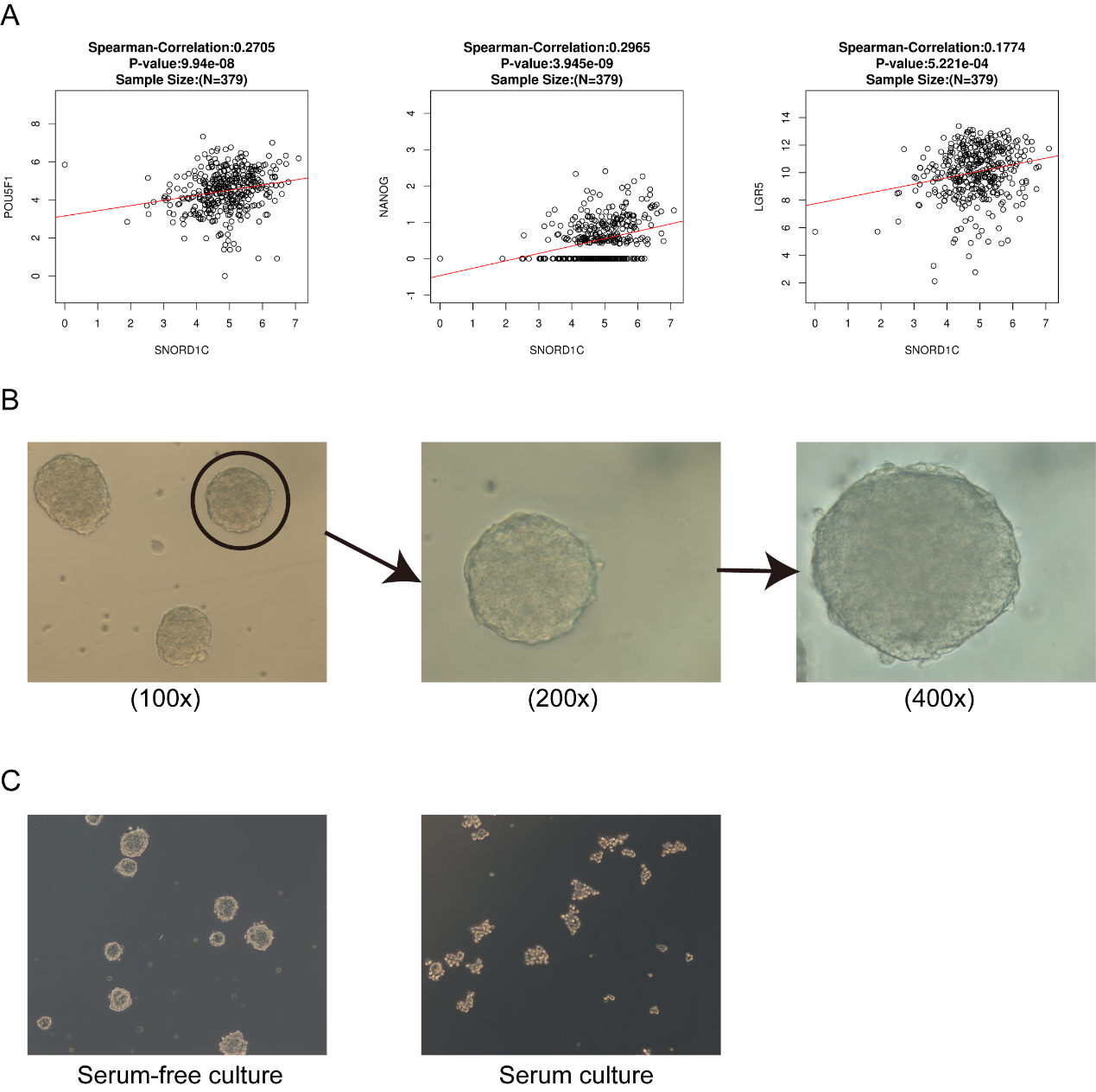


**Fig. S2** **SNORD1C had the potential to regulate and promote stem cell formation. A** Correlation analysis of SNORD1C and OCT4, NANOG, LGR5 in TCGA database. **B** Tumor spheroid cells were formed in SW620. **C** Serum-induced differentiation of tumor spheroid cells in SW620.
