# Supplementary material for: SNORD1C maintains stemness and 5-FU resistance by activation of Wnt signaling pathway in colorectal cancer
Source: Cell Death Discov. 2022 Apr 14;8:200. doi: 10.1038/s41420-022-00996-5 (PMC9010412; doi:10.1038/s41420-022-00996-5)
Supplement: Supplementary file 2 — Original western blots [file 41420_2022_996_MOESM2_ESM.docx]

_­_­ **SNORD1C maintains stemness and 5-FU resistance by activation of Wnt signaling pathway in colorectal cancer**

**Figure 4**

- Fig.4G:


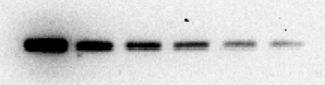


β-catenin


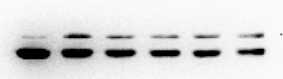


GAPDH

- Fig.4H

**SW620:**


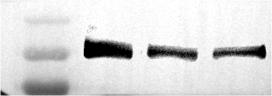


β-catenin


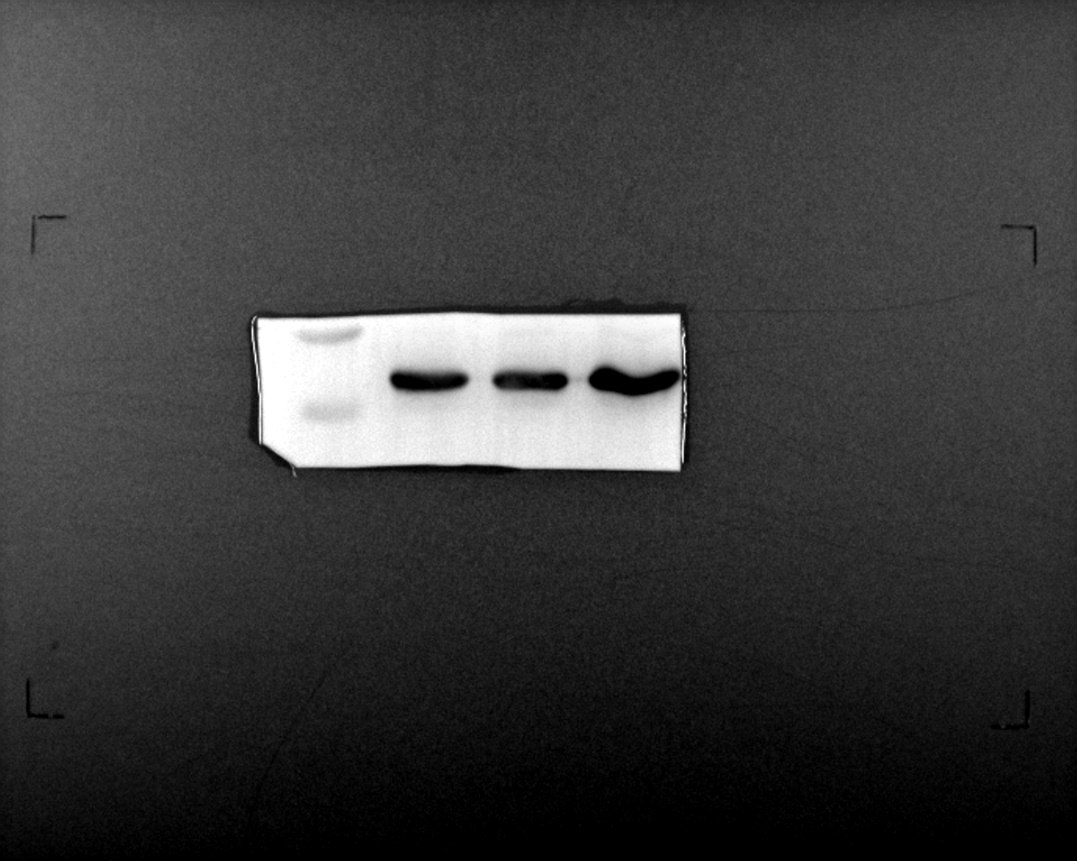


GAPDH

**SW480:**


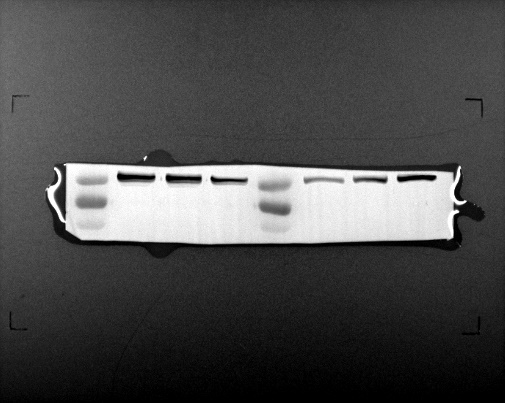


β-catenin


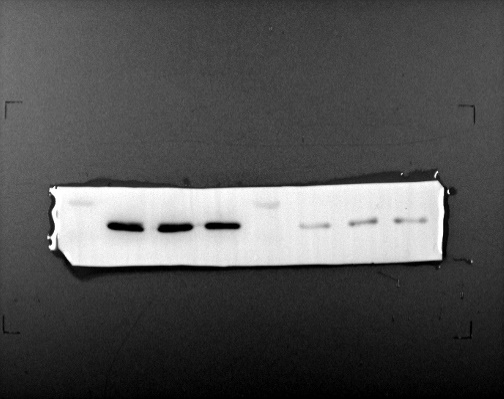


GAPDH

- Fig.4J

**SW480(nc, si-1, si-2); SW620(nc, si-1, si-2):**


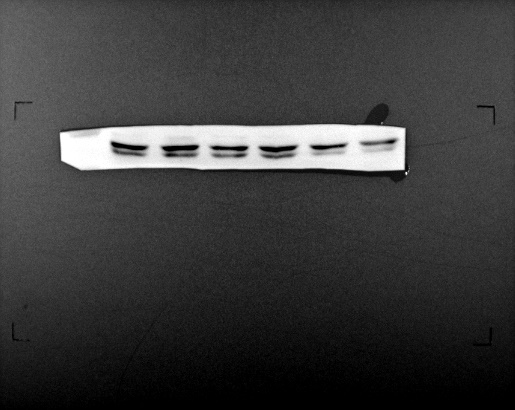


TCF7


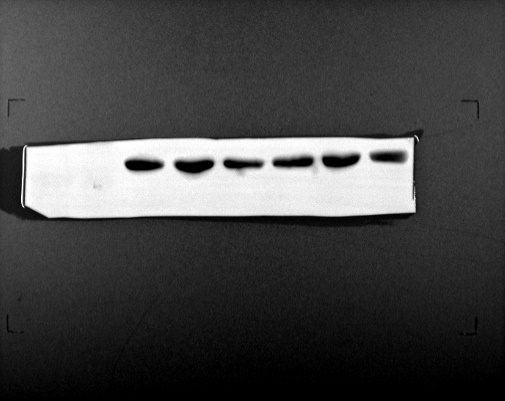


GAPDH

**HCT116 (vector, SNORD1C-pcDNA):**


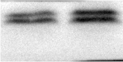


TCF7


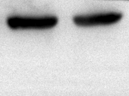


GAPDH

**DLD1(vector, SNORD1C-pcDNA)**


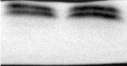
TCF7


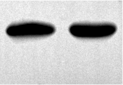
GAPDH

**Figure 5**

- Fig.5B


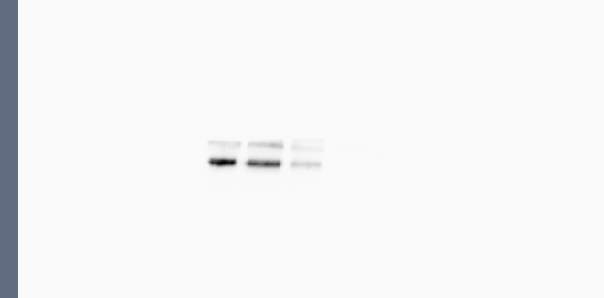


CD44





GAPDH

- Fig.5C


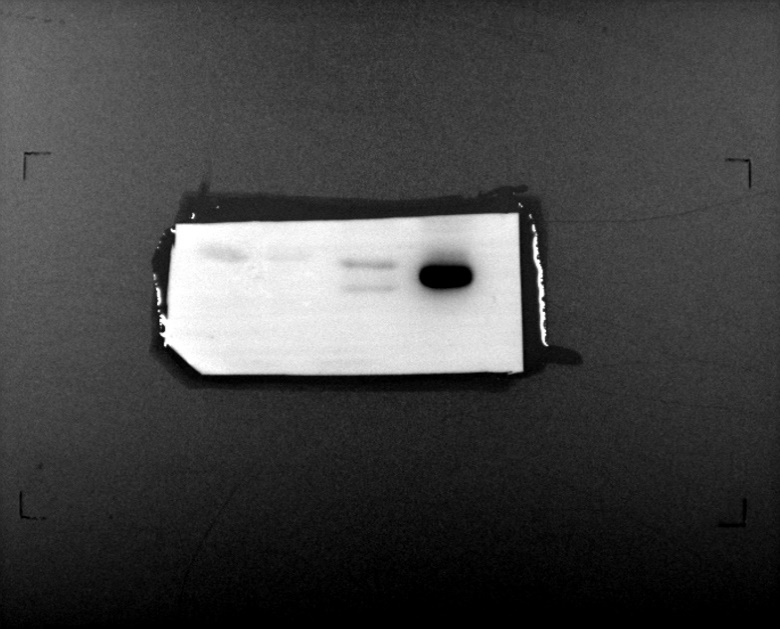


SOX2


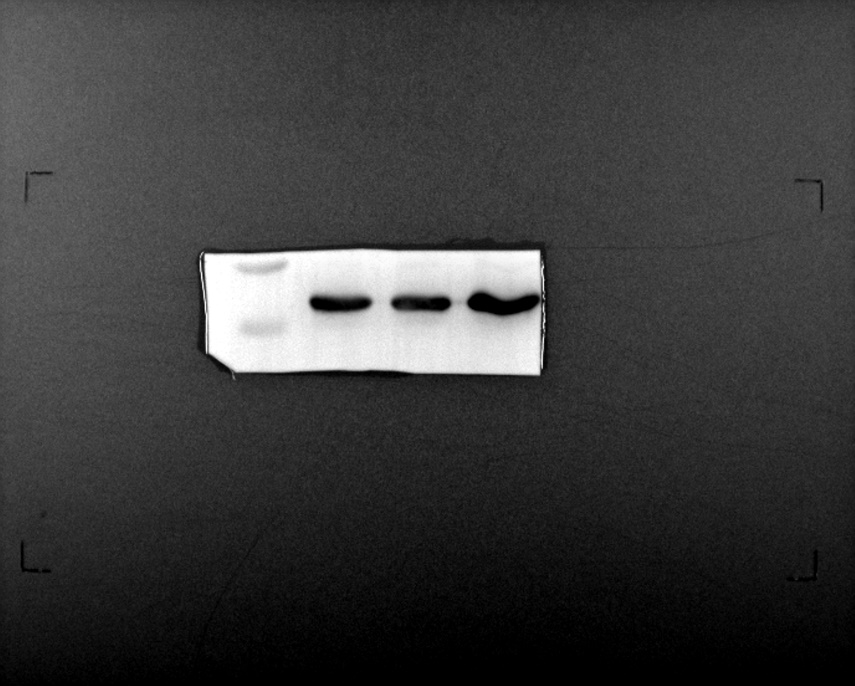


GAPDH
